# Supplementary material for: Age and sex-related variations in murine laryngeal microbiota
Source: PLoS One. 2024 May 14;19(5):e0300672. doi: 10.1371/journal.pone.0300672 (PMC11093383; doi:10.1371/journal.pone.0300672)
Supplement: Attachment — Submitted filename: Response letter_0201st.docx [file pone.0300672.s001.docx]

**January 17^th^ 2024**

Peter Gyarmati
Academic Editor, *PLOS ONE*

Dear Dr. Gyarmati,

We would like to thank the reviewers for the valuable feedback on our manuscript. We are pleased that the reviewers found this manuscript “well-designed” and “interesting.” In incorporating reviewer feedback, we believe that the impact, organization and clarity of this manuscript is much improved. We are thankful for the opportunity to revise and resubmit. Please find our responses to each of the editor and reviewer’s comments below.

**Clarification of Funding and Funder role**

As per the editor’s request, we would like to clarify that “the funders had no role in study design, data collection and analysis, decision to publish, or preparation of the manuscript.” Our study was funded by the National Institute of Deafness and other Communication Disorders, National Institute of Health (NIDCD, NIH). We have made sure that funding information in the ‘Funding Information’ and ‘Financial Disclosure’ sections match.

**Editor**

1. *Asks authors to reformat manuscript and figures to PLOS ONE requirements***.**

**RESPONSE:** We have complied.

2. *Asks authors to deposit original data in a repository.*

**RESPONSE:** 16S rRNA gene sequencing data are accessible in GenBank under BioProject PRJNA1041457.

3. *Requests authors to add details to methodology regarding (1) methods of sacrifice, (2) methods of anesthesia and analgesia and (3) efforts to alleviate suffering.*

**RESPONSE:** Animals did not undergo any experimental procedure, thus, anesthesia, analgesia or measures to alleviate suffering were not employed in the present study. We have included additional details for our euthanasia procedure as follows; For the experiment, mice were sacrificed via CO_2_ inhalation in L103.

4. *Requests clarification of data sharing plan.*

**RESPONSE:** As stated above, 16S rRNA gene sequencing data has been deposited in GenBank under BioProject PRJNA1041457 and will become available on acceptance. We also included a Data availability statement in the manuscript, L397.

5. *Add an ethics statement to the Methods section of the manuscript.*

**RESPONSE:** We have complied**.** We have clarified our ethical review board approval with the	following statement “All procedures and protocols were approved by the Institutional Animal	Care and Use Committee (IACUC) at University of Wisconsin-Madison (Protocol: M005669).

This can be found in L94.

**Reviewer 1**

We thank the review for the insightful comments. We have made changed accordingly. Please note that line number may have changed for some comments after revision.

1. *Suggests expansion and clarification of results and figures.*

**RESPONSE:** We thank the reviewer for this suggestion. We have incorporated the following changes based on the reviewer’s suggestion: (1) age category names that are associated with each group, (2) Figure 4 was edited to incorporate significant labels (3) Figures 5 and 6 now have further explanations, (4) added Figure 6B results to the main text (L210-213), (5) expanded on the statistical tests incorporated, (6) edited Figure 1 to match the text.

2. *Asks for clarification for the analyses for sex-based differences (to remove confounding age effects.*

**RESPONSE:** We have incorporated a Lar model with the data (using the *limma-voom* function in *limma* package in R) to remove the age effect and have included the corresponding results in the methods (L161), results (L275), and discussion (L361) of the manuscript.

3. *Requests additions to the discussion.*

**RESPONSE:** We have incorporated all the reviewer’s suggestions. Specifically, we have added a paragraph describing the study limitations and future directions (L368). We have also age-related changes in the upper airway in the discussion (L347).

4. *Suggests the following changes to improve readability.*

1) *Correct typos (including double parenthesis, using period instead of commas) and clarify information about the gut microbiome in the introduction.*

**RESPONSE:** We have corrected all typo throughout. We have altered our discussion of the gut microbiome in the introduction to increase clarity (L57-67).

2) *L296 has a missing word.*

**RESPONSE:** We have corrected this sentence.

3) *L96-97 requires clarification on the species.*

**RESPONSE:** We have complied.

4) *Methods include versions of software when relevant.*

**RESPONSE:** version has been added to each software used in the manuscript.

5) *Correct L167-168 to show that 1 adolescent and 1 mature adult was removed.*

**RESPONSE:** We have corrected this sentence, and now it’s in L153.

6) *Incorporate the full form of ASV (amplicon sequence variant) at the start of the new section.*

**RESPONSE:** We have complied, which is in L144.

7) *Remove double parenthesis in L186-188.*

**RESPONSE:** We have complied.

8) *Correct paragraph 1 of the results (L181) as authors call the same group two names (“middle-aged”, “older adult”).*

**RESPONSE:** We have complied and corrected L185 (line number changed after revision) to address this group consistently as “older adults”

9) *Move L189-190 to the discussion.*

**RESPONSE:** We have complied.

10) *Clarify whether L203-204 refers specifically to Corynebacterium.*

**RESPONSE:** We have complied (L204).

11) *Clarify whether “groups” in L205 refers to sex.*

**RESPONSE:** We have complied.

12) *Clarify the statistics in L207-208.*

**RESPONSE:** We have complied. We add more detailed statistics to the Methods section (Welch’s t-test, L166).

13) *Write out the full form of LEFSe,*

**RESPONSE:** We have complied.

14) *Move L248 to the discussion.*

**RESPONSE:** We have complied.

15) *Remove the word “confirmed” in L318.*

**RESPONSE:** We have complied.

16) *Remove the journal name when citing a manuscript in review.*

**RESPONSE:** We have complied.

17) *Data availability statement should be placed with the acknowledgements.*

**RESPONSE:** We have complied (L397).

**Reviewer 2**

1. *Asks for clarification on how microbial changes could potentially drive atrophy in presbylarynx.*

**RESPONSE:** We thank the reviewer’s insightful comment. We agree that understanding how microbial changes could potentially drive the atrophy in presbylarynx or other diseased conditions is important and beneficial. As a first step towards the long-term goal, this study intends to delineate age-related variation in the resident microbiota (normal flora) in the larynx of conventionally raised mice. It is, to the authors’ knowledge, the first study to investigate the temporal stability of laryngeal microbiota across the lifespan in any model (murine or human). We have on-going projects in the lab along this line of research, where germ-free mice are colonized with a single or mixed bacterial strains to delineate how specific microbial variation influence host laryngeal physiology.

2. *Requests expansion of age-related changes of microbiota in other parts of the airway in the Introduction.*

**RESPONSE:** We appreciate this reviewer’s comment. Microbiota in other parts of the airway (oral and nasal) have a similar lack of literature delineating age-related variation. In the oral microbiota, *Neisseria* is reduced, and *Streptococcus anginosus* and *Gemella sanguinis* are increased with age (Kazarina et al., 2023), which are not observed in the laryngeal microbiota. Thus, more research is required to compare similarities and differences in all upper airway microbiota across the lifespan. As found in the laryngeal microbiota, oral microbiota also show high variation in microbial composition across the lifespan (Bach et al., 2020). We have incorporated this in the manuscript, which can be found in L68, 71,74.

3. *Suggests an expansion of model limitations (specifically the lack of mechanical stresses from vibration and the environment that exist in the murine model)*

**RESPONSE:** We have added the following to the limitations section as per this reviewer’s suggestion; “In addition, murine laryngeal microbiota exhibited stability with age and sex. However, we acknowledge that environmental differences, as well as mechanical stresses from vibration, differentiate the human model from the murine model. These factors may contribute to aging related findings and cannot be replicated in the murine model. Thus, future work should investigate whether similar age and sex-related variation exist in the human laryngeal microbiota. We have addressed these limitations and next steps in the manuscript”. This can be found in L378.

4. *Asks for correction of typos.*

**RESPONSE:** We have carefully revised the manuscript and corrected all typos.
